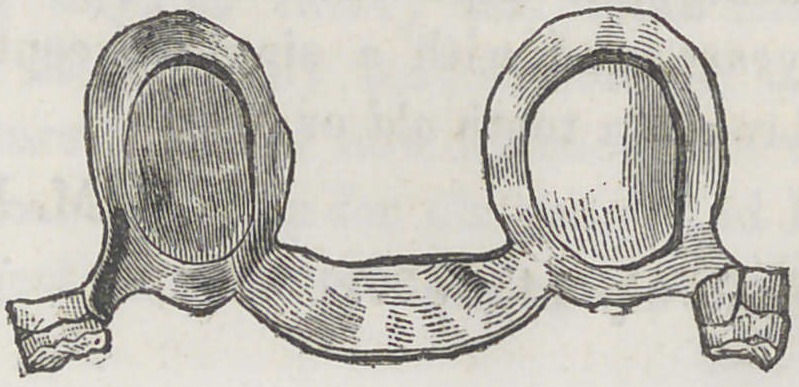# Pressure Plate

**Published:** 1859-09

**Authors:** J. Taft


### PRESSURE PLATE.

BY J. TAFT.

FORMERLY it was almost the universal practice, to attach partial sets of teeth by clasps ; within the last few years, however, an increasing effort has been made, to devise some method of retaining such pieces without the aid of clasps. The only method available for the accomplishment of this purpose is atmospheric pressure plates. In the great majority of cases, with the improvements now employed, this can be accomplished; ordinarily this is done by forming a plate, generally with a chamber, that shall cover a considerable portion of the palatal arch. Under ordinary circumstances, when four, six, or eight teeth are to be supplied, this arrangement is available, but when there are one or two teeth to be supplied at each side—the bicuspids for instance—there is frequently considerable difficulty experienced in arranging an ordinary atmospheric pressure plate; pressure upon one side alone, has a tendency to detach the plate at the other. I have recently had occasion to insert the second superior bicuspid upon each side; it was desirable to avoid the use of clasps, and yet avoid the use of a plate that would cover a large portion of the arch. A cavity plate about the size of a dime, was made for each tooth, and the tooth attached in proper position, the plate resting on the side of the arch; in juxtaposition to the space to be occupied by the tooth, these two plates are attached together in proper relative position, by a band of gold about two lines in width, passing from one to the other, from two to three lines posterior to the inner portion of the front teeth; this strip is swedged so as to fit the arch perfectly, it serves to keep the plates in place, and by it, each one sustains the other to some extent.

This arrangement serves a valuable purpose, much better, I think, than the common single cavity plate would do in the same place, and with not more than one-third the amount of gold. The same principle would be equally applicable for the insertion of two or three teeth upon each side. The following cut represents fully the piece above described, and this in connection with what has been said, will give a perfect idea of the plan.

## Figures and Tables

**Figure f1:**